# Splitting of a Dexamethasone Implant (Ozurdex) following the Injection

**DOI:** 10.1155/2013/247949

**Published:** 2013-07-30

**Authors:** Oya Donmez, Melih Parlak, Aylin Yaman, Ali Osman Saatci

**Affiliations:** ^1^Department of Ophthalmology, Dokuz Eylul University, Mithatpasa Cad, 35340 İzmir, Turkey; ^2^Department of Ophthalmology, Klinikum Konstanz, Luisenstraße 7, 78464 Konstanz, Germany

## Abstract

In this brief report, we share our observations on a splitted Dexamethasone implant (Ozurdex) which we discovered a week after the injection. It is likely that implant splitting neither changes the efficacy of the implant nor creates a mishap for the patient.

## 1. Introduction

 Dexamethasone implant (Ozurdex) is approved for eyes with posterior uveitis and macular edema associated with retinal vein occlusions [1, 2]. Very recently, splitting of the implant following the injection was reported in two papers [3, 4]. We hereby report our observation on a splitted Dexamethasone implant which was discovered a week after the injection.

## 2. Report of a Case 

**Figure 1:**
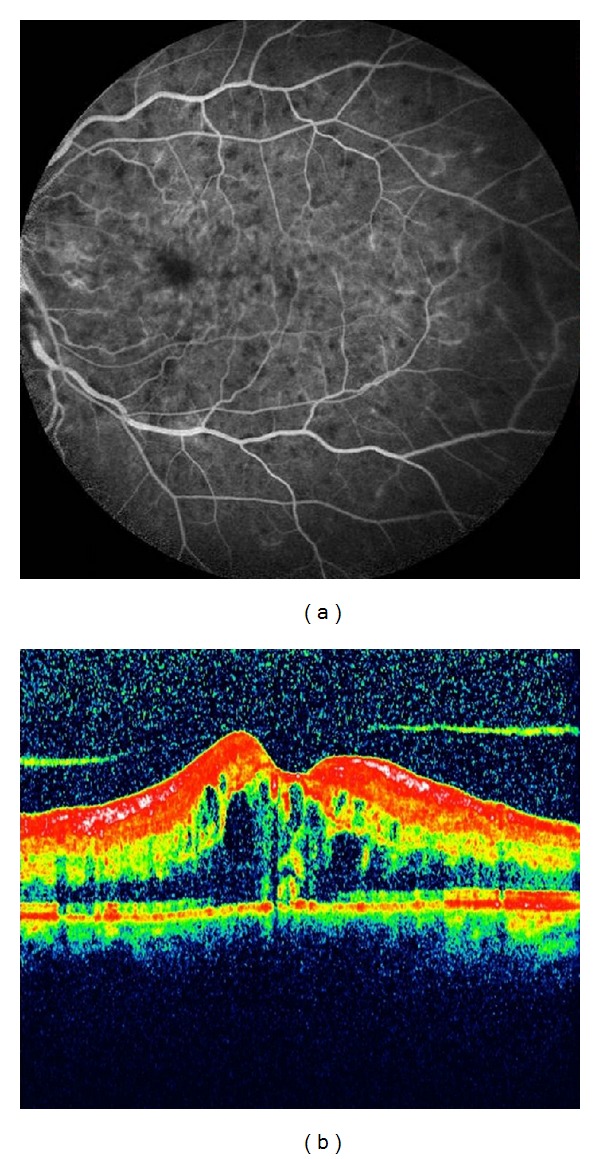
(a) Angiographic picture of the left eye depicting the central retinal vein occlusion and macular edema. (b) Optic coherence tomography showing the macular edema and attached vitreous in the left eye.

**Figure 2:**
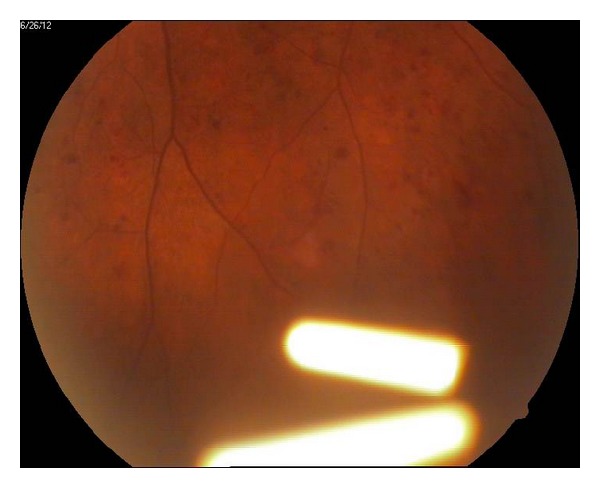
Splitted Ozurdex implant in the lower part of the vitreous.

**Figure 3:**
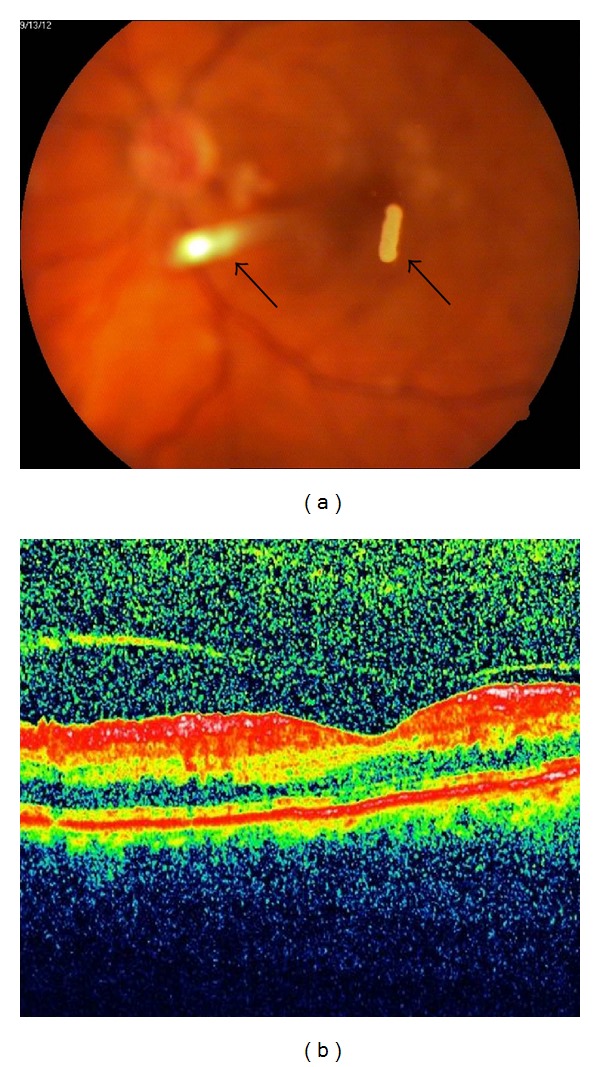
(a) Colour fundus picture at the third postinjection month showing the partly dissipated splitted Ozurdex implant (arrows). (b) Optic coherence tomography demonstrating the resolution of macular edema at the third postinjection month.

 A 68-year-old man received a Dexamethasone implant (Ozurdex) for macular edema due to nonischemic type central retinal vein occlusion at our institution (Figures 1(a) and 1(b)). The implantation was performed according to the advised conventional way. We do not routinely examine the fundus immediately after the implant injection. At the first visit, on day seven, we noticed that the implant was in the lower part of the vitreous in two pieces (Figure 2). His visual acuity was improved to 20/40 from 20/100 and the macular edema subsided. Macula remained free of edema and splitted Ozurdex fragments were visible in front of the macula at the third postinjection month (Figures 3(a) and 3(b)). No injection-related complication occurred during the followup. 

## 3. Discussion

 The implant is preloaded into a specially designed single use applicator that promotes easy and controlled drug delivery. Thereby, the slow release provides long-term effectivity and less steroid-related side effects. First, Rishi et al. [3] reported that the Ozurdex implant broke into two pieces immediately after the injection. Possible causes would be (i) implant got cracked during manufacturing/packaging and broke during injection, being unable to endure the force of injection process and (ii) possible misalignment of implant within the injector, leading to shearing forces breaking it during injection. Roy and Hedge [4] reported in a patient with diabetic macular edema that injected Ozurdex implant was splitted into two pieces immediately after the injection. The authors speculated that the implant split might possibly alter the drug release with relatively higher initial drug concentration and faster dissipation. In the present case, the effect of splitting implant was not altered and we did not experience any side effect due to implant breaking.

 Implant splitting may not be a rare occurence but Ozurdex splitting most possibly do not change the effectivity of the implant or create a mishap for the patient.
